# Supplementary material for: Are carbon emissions trading and green financial instruments synergistic? -Comprehensive quantitative research based on content analysis
Source: PLoS One. 2024 Mar 7;19(3):e0298601. doi: 10.1371/journal.pone.0298601 (PMC10919601; doi:10.1371/journal.pone.0298601)
Supplement: S1 Data — (ZIP) [file pone.0298601.s001.zip › Data/地级市Z_F工作报告.zip!/╣π╬≈/╣≡┴╓╩╨/└┤╫╘╣1⁄2╓┌║┼í╛┬φ┐╦╩2╛▌═°í┐_2015.docx]

各位代表：

　　现在，我代表市人民政府向大会作工作报告，请予审议，并请各位政协委员和列席会议的同志提出意见。

　　一、2014年工作回顾

　　2014年，面对复杂的经济形势和艰巨繁重的改革发展稳定任务，面对“三期叠加”[1]带来的诸多挑战，我们在自治区党委、政府和市委的正确领导下，紧紧围绕市四届人大五次会议确定的目标任务，主动适应经济发展新常态，坚持稳中求进工作总基调，坚持建设桂林国际旅游胜地“一本蓝图绘到底”，全力以赴稳增长、促改革、调结构、惠民生、防风险，全市经济社会呈现稳中有进、稳中提质、稳中向好的发展态势。初步核算，地区生产总值1827.1亿元，增长8%，其中第一产业增加值323.1亿元，增长4.9%，第二产业增加值865.1亿元，增长9.9%，第三产业增加值638.9亿元，增长6.6%；组织财政收入195.2亿元，增长8.2%；全社会固定资产投资1645.3亿元，增长18.3%；社会消费品零售总额682.9亿元，增长13.1%；城镇居民人均可支配收入26811元，增长9.2%，农民人均纯收入9431元，增长12.8%；居民消费价格涨幅2%；城镇化率达到45.6%；万元地区生产总值能耗下降3.5%，减排指标完成自治区下达任务。

　　一年来，我们主要做了以下重点工作：

　　(一)实施项目带动战略，旅游胜地建设实现新突破

　　以加快重大项目建设为抓手，推动旅游产品、旅游管理及旅游业态转型升级。全面推行项目建设“五个一”和“三清单一问责”机制，开辟重大项目并联审批“绿色通道”，加快项目落地及建设速度。全年实施重大项目883项，完成投资793.8亿元，市领导包抓的20个重中之重项目和38个重大项目进展顺利。一批大型知名商业综合体建设开历史先河，高新万达广场封顶，华润万象城、海吉星食尚港启动建设，国际商贸城、万福休闲广场基本建成。一批文化、旅游及服务业项目建设强力推进，保利文化产业园、瑞盛旅游休闲世界、信和信·桂林国际智慧健康旅游产业园等项目进度加快，国际知名酒店品牌悦榕庄阳朔度假酒店建成营业，胜地建设支撑能力进一步增强。加强汇报，积极跟踪，政策争取和落地力度大、效果好。51国外国人72小时过境免签和东盟10国旅游团6天入境免签获得国家批复，旅游产业用地改革试点政策落地实施，桂林成为全国首获上述政策的地级市，经济发展空间进一步扩大，动力更加强劲。旅游新业态不断拓展，品质不断提升。航空旅游、高铁旅游齐头并进，机场旅客吞吐量接近700万人次，增长17.6%；铁路旅客发送量达720万人次，增长66%。与海航集团共同组建桂林航空旅游集团有限公司，打造“航空+旅游”“旅游+金融”“智慧旅游”三大经济发展引擎。“空中游漓江”试飞成功，填补广西高端航空旅游项目空白。地中海俱乐部桂林愚自乐园度假村、罗山湖、玉圭园等高端休闲旅游产品获得市场认可。休闲游、特色游、体验游持续兴旺，自驾游、自助游涨势迅猛，旅游逐步向多元化和休闲度假模式转变。旅游品牌影响力不断增强。旅游服务业标准化试点城市建设通过国家验收，特色旅游名县创建工作全区领先，阳朔、兴安荣获首批广西特色旅游名县；桂林喀斯特地貌列入世界自然遗产名录。成功举办第八届联合国世界旅游组织/亚太旅游协会旅游趋势与展望国际论坛，桂林成为国际旅游数据研究与趋势展望的“智慧中心”之一；桂林国际旅游博览会获全国金五星优秀旅游展会奖。与阿里巴巴合作搭建桂林—淘宝旅游合作平台，与百度合作设立“@桂林旅游”直达号，成为全国首个百度直达号旅游线上到线下城市。全年接待游客3871.2万人次，增长8%，其中入境游客203.3万人次，增长5%；旅游总收入420.3亿元，增长20.6%；国内游客、入境游客人均停留时间分别达2.77天和2.15天；游客人均消费突破1000元，增长11.6%，其中入境游客达2928元/人次。旅游总收入、游客人均消费增幅明显高于游客数量增幅，旅游转型升级效果显现。

　　(二)调转并举稳中提质，产业转型升级凸显新成果

　　工业经济优化提升。出台支持工业发展的系列政策措施，实施“企业直通车“千名干部入千企”等服务制度，努力破解企业发展难题。完成工业投资621.1亿元，增长13.6%；规模工业增加值657.7亿元，增长10.6%；规模工业总产值2116.7亿元，增长10.9%，其中电子信息、医药及生物制品、先进装备制造、生态食品四大重点产业产值突破1000亿元，工业对经济贡献率达55.2%。以高新区为主体的国家新型工业化(电子信息)产业示范基地建设稳步推进，临苏经济开发区规划编制及全市园区管理体制机制改革有序推进。百强企业对规模工业增长贡献率达76.6%，国际电线电缆集团等3家企业入围中国机械工业500强，漓泉等3家企业成为首批国家两化融合管理体系贯标试点企业，优利特等3家企业成为国家火炬计划重点高新技术企业，福达股份获自治区主席质量奖，企业核心竞争力不断提升。

　　现代农业加快发展。实现农林牧渔业总产值514.1亿元，增长5%。粮食总产量达206.2万吨，蔬菜、肉类、水产品产量稳定增长，水果、食用菌、竹狸、冷水鱼总量全区第一，牛、羊等草食动物增势迅猛，恭城成为国家级出口水果质量安全示范县，平乐成为国家农作物种植综合标准化示范区，灌阳获全国粮食生产先进县。桂林国家农业科技示范园区启动建设。建成推出4个现代特色农业(核心)示范区，成为全区先进典型。休闲农业走在全国前列，乡村旅游收入增长20.6%，龙胜成为全国休闲农业与乡村旅游示范县。新增市级以上重点产业化龙头企业40家、农民专业合作社566家。川江水库下闸蓄水，小溶江水库主体工程基本完成，斧子口水库加快推进，完成68座病险水库除险加固，新增、恢复和改善灌溉面积39.4万亩；新增100个建制村通水泥(沥青)路，建制村通畅率达83%，新增通自然村屯公路290公里。373个村屯安装太阳能路灯，完成1650个村屯内3087公里道路硬化，400个“普惠制”新村完成投资3.26亿元。深入实施农民收入倍增计划，切实落实各项支农惠农政策，发放各种惠农资金21.3亿元。

　　服务业提质发展。新增限额以上服务业企业103家，位居全区前列，服务业对地方财税贡献率达64%。积极应对政策性因素的影响，推出和落实促进健康养生、房地产等服务业发展的政策措施。加强服务业标准化建设，开展第一批18家星级米粉店等级评定工作。培育电商、动漫等新业态，与国家信息中心开展信息规划、产业、惠民等方面的深度合作，桂林“电商谷”入驻企业近300家，力港网络科技公司入选第六批国家文化产业示范基地，基于互联网的信息消费成为新热点。获批创建国家电子商务示范城市、国家信息惠民试点城市。全市金融机构存款余额2269.8亿元，贷款余额1389.6亿元，总量和增量均居全区前列。成功争取中国—东盟博览会旅游展和广西名特优农产品交易会永久落户桂林，成功举办第二届中国(桂林)国际健康养生服务产业创新发展高端论坛及首届进口商品交易会暨海峡两岸名品展，荣获全国优秀会展城市奖。

　　创新能力明显增强。全年发明专利申请3670件，每万人拥有发明专利3.11件，均居全区第一。荣获1项国家技术发明奖，囊括2项广西科学技术特别贡献奖。桂林大学科技园成为广西首家国家级大学科技园，桂林理工大学科技园成为自治区级大学科技园。与“985”高校合作取得实质性进展。桂林国家级文化和科技融合示范基地建设加快推进。三金药业跨入国家级创新示范企业行列。

　　(三)建治并重统筹推进，城乡建设呈现新面貌

　　区域性交通枢纽建设步伐加快。立体交通网络建设取得实质性突破。两江机场T2航站楼开工建设；覆盖我市8县的灌阳(永安关)至全州(凤凰)、阳朔至鹿寨、资源至兴安、桂林至三江等4条高速公路加快推进，在建里程282公里，完成投资44亿元，均创历史新高；桂林北站改造和桂林西站新建工程全面完成，贵广高铁建成通车，全市高铁通车里程达420公里，占全区高铁通车总里程近三分之一，成为国内首个“一市九站两高铁”[2]的地级市，桂林全面融入四省会3小时经济圈，开启了高铁经济发展新征程。

　　新老城区功能不断完善。临桂新区实施建设项目79项，完成投资52亿元。“一院两馆”等一批标志性项目竣工使用，桂林广电大厦顺利封顶；宏谋大道、山水大道等主要路桥工程扎实推进，“五纵三横三环”[3]路网结构基本成型；商务、文教、医疗等配套设施及公交、管网、绿化等市政设施加快完善。市直机关正式入驻，新区人气不断聚集，活力日益彰显，基本实现“五年初见成效”目标。深入实施连接新区老城交通大动脉的“南畅、北通、东拓、西联”工程，国道321阳朔至桂林段扩建工程全面动工，滨江北路全线通车，西二环路基本达到通车条件，福利路二期、万福东路、龙门大桥等新建工程开工建设，市区交通路网进一步完善。虞山片区、福隆园片区、下关塔山片区等棚户区改造工程启动，滨水生态广场、正阳东西巷历史文化街区等项目主体工程完工，旅游厕所建设提升工程加快推进，城市功能品位进一步提升。县城和小城镇建设持续推进，临桂县城与新区实现融合发展，灵川与桂林同城化步伐加快，全州、荔浦新城区初具规模，灌阳、平乐、阳朔等县城新区建设取得新进展，资源、龙胜、恭城、兴安、永福等县城基础设施建设和风貌改造迈出新步伐。第一批13个新型城镇化示范乡镇验收命名，6个“广西特色名镇名村”建设力度加大，城乡风貌改造完成投资6882万元，人居环境持续改善。

　　城乡环境综合整治成效显著。按照“精、细、严、新、实”的总体要求，强力推进环境综合整治，全力做好重拳治堵、重点治脏、切实治乱等重点工作，一批市民反映强烈的城市管理“老大难”问题加快解决，城市交通更加有序，街道更加干净，市民更加文明，胜地形象更加靓丽。优化交通组织，坚持惩罚教育并重，规范停车秩序，全力疏堵治乱，交通拥堵有效缓解。主城区新建或改造整治了一批停车场、集贸市场、垃圾转运站、公共厕所及无物业管理小区和城中村。运用行政、法律、市场等手段，着力推进环境卫生综合管理，中心城区环卫保洁基本实现精细化，无物业管理小区、城中村、城乡结合部环卫保洁基本全覆盖。以严控新的违法建筑为突破口，强力推进“控违”“拆违”专项整治行动。持续开展创建全国文明城市活动。“智慧城市”建设顺利推进，地下综合管线信息管理平台投入使用。推动“美丽桂林·清洁城乡”活动向纵深开展，17个村屯获自治区“清洁乡村·百佳村屯”称号，新增自治区级生态乡镇14个，列全区第一。持续开展农村环境连片整治。加强漓江生态和饮用水源保护，建立完善青狮潭水库长效管理机制，水库水质明显改善，主要河流监测断面水质达标率100%。我市成为第二批全国水生态文明建设试点城市。继续实施“绿满八桂”工程，生态环境持续优化。高度重视大气污染防治工作，启动对大气质量有严重影响的小砖厂、小锅炉、采石场、汽车尾气、施工工地及道路扬尘等污染源治理工作，市区环境空气质量优良率达68.5%，比上年提高4.4个百分点。严格落实耕地保护制度。有序推进低碳城市试点。规模工业万元增加值能耗下降16.7%，超额完成自治区下达任务。

　　(四)深化改革扩大开放，发展内生动力得到新增强

　　重视对各项改革的制度设计和具体推动，着力破解影响经济社会发展的不合理的体制机制。政府机构改革步伐加快，城市管理体制、漓江管理体制、国有企业监管体制、服务业统计和工商登记制度等改革有效推进。出台政策促进非公经济跨越发展。拓展融资渠道，推动市场化多元融资，探索融资租赁、股权融资等融资新模式，与上交所建立资本市场合作机制，福达股份在上交所主板成功上市，瀚特公司在“新三板”成功挂牌，融资工作打开新局面。农村土地承包经营权流转、集体林权制度配套改革进展顺利，新增土地流转12.4万亩、林地流转24万亩，农村集体土地确权登记颁证工作取得阶段性成果。全面启动县级中等专业学校综合改革，我市被确定为全国特殊教育改革试验区。加快县级公立医院管理体制、补偿机制、医保支付方式等改革，10个县纳入县级公立医院综合改革国家级试点。坚持“选资择商招大引强”，一批“高大上”项目相继落户，新签市外境内项目总投资632.1亿元，市外到位资金625.8亿元，实际利用外资4.37亿美元，成功引进一批强企名企。与张家界等多个城市签署战略合作协议，与法国阿讷西等城市加强友好交流。引导企业加强对外经济合作，企业“走出去”取得实效，桂林矿产地质研究院控股子公司并购加拿大奥斯汀资源有限公司在多伦多复牌上市，广西师大出版社成功并购澳大利亚视觉出版集团。创建国际卫生机场工作有序推进。完成外贸进出口总额9.43亿美元，其中出口7.72亿美元。

　　(五)加强保障改善民生，社会事业发展再上新台阶

　　坚持民生为先，努力使发展成果惠及全体市民，全市公共财政民生领域支出228.8亿元，占预算支出的73.6%，自治区级和市级为民办实事工程全面完成。全市发放城乡最低生活保障补助资金5.86亿元，城镇新增就业7.01万人，农村劳动力新增转移就业7.52万人次，城镇登记失业率3.8%，城乡居民养老保险参保率98.75%，新农合参合率99.46%。新开工建设各类保障性住房9516套，基本建成保障性住房11057套，分配入住6703套，改造农村危房18897户，解决农村34.3万人饮水安全问题。稳步推进城乡公交一体化建设，全市乡镇、建制村通公交率分别达45.1%和28.2%，受益群众超过130万人。公共自行车服务系统投入使用。投入各类扶贫资金8亿多元，实施扶贫项目1005个，减少贫困人口7万人。实施“寻找桂林文化的力量，挖掘桂林文化的价值”工程，成立桂林历史文化研究院，历史文化资源调查工作顺利完成，甑皮岩国家考古遗址公园正式挂牌，成为华南地区首个国家考古遗址公园；“飞虎队”桂林遗址公园纪念馆主体工程完工；桂林渔鼓、资源河灯歌节、龙胜红瑶服饰入选第四批国家级非物质文化遗产名录。社会主义核心价值观系列动漫公益剧《可可小爱》成为全国动漫业界获奖最多的单个动漫品牌，并在中央电视台、中国文明网等众多媒体播放；“百姓大舞台”等公益性文化活动影响力持续提升。实施教育发展十大重点工程，城区新建扩建8所义务教育学校，学前教育三年毛入园率、九年义务教育巩固率、高中阶段教育毛入学率均居全区前列，高考一本上线率全区第一，资源、龙胜、临桂、阳朔成为全区第一批实现12年免费教育县，完成市卫生学校等8所中等职业学校的归口管理。1348所村卫生室实施乡村卫生服务一体化管理，覆盖面达81%，成功举办桂林中医文化节。落实单独二孩政策，诚信计生实现率98.6%，人口自然增长率6‰。全民健身运动持续开展，完成第四次国民体质监测，成功举办世界女子九球锦标赛，桂林籍运动员获第17届仁川亚运会男子吊环冠军。落实安全生产责任，加大隐患排查整治，安全生产形势持续稳定，兴安、资源及恭城荣获全国“平安农机”示范县。食品药品监管得到加强，应急处置能力有效提升。实施市县区领导包抓化解突出信访积案制度，一批信访积案按时化解办结，群访和越级上访人次明显下降。深入实施“六五”普法，开展净化社会治安环境大整治专项行动，发案量创五年新低，平安桂林建设成效显著，社会保持和谐稳定。

　　（六）转变职能提高效能，政务服务环境得到新改善

　　扎实开展党的群众路线教育实践活动，努力构建集学习政府、改革政府、服务政府、廉洁政府、责任政府、法治政府为一体的政府运行模式，依法行政水平不断提高，政务环境不断优化。创新学习模式，健全学习制度，开展“市长上讲台”活动，去年5月以来，市政府领导班子成员18次走上讲台授课。加强行政审批制度改革，全力抓好行政审批“接、放、管”，市本级取消、下放、承接、调整行政审批项目29项，清理上报拟取消、下放和调整行政审批事项36项；优化审批流程，实行“马上办”工作机制，涉及市本级205个审批服务事项实现马上办结，53.8%的审批服务实现窗口现场审批，即办件量从新机制实行前的月均910件增加到5398件，行政审批效率平均提速66.8%。切实推进行政权力网上公开、政务公开和政府信息公开。认真落实市人大及其常委会决议决定，接受市人大、市政协的工作监督、民主监督，人大代表建议、政协提案办结率100%，满意率均达98%以上。充分发挥“大督查”机制作用，强化政府工作督查。整治“四风”突出问题，市本级“三公”经费支出减少31.2%，全市性会议减少30%；着力查处“吃拿卡要”“庸懒散拖”等问题，廉政建设有效加强，干部作风明显好转，行政效能不断提高，政府公信力进一步增强。

　　一年来，外事、侨务、对台、审计、统计、工商、税务、海关、检验检疫、海事、通信、邮政、高校院所、新闻广电、移民、地震、人防、消防、保密、测绘、气象、水文、发展研究、机关事务、供销、地方志、社会科学、档案、宗教、老龄、残疾人、妇女儿童等工作取得新进展。国防教育和后备力量建设、双拥共建工作不断加强，11个民族乡乡庆活动成功举办，民族团结进步创建活动深入开展，中直、区直驻桂单位为桂林发展出力献策，取得新成绩。

　　各位代表，过去的一年，是“十二五”以来发展形势最为复杂的一年，面对持续加大的经济下行压力，全市上下精准发力，应对给力，凝心聚力，大家确实蛮拼的。虽然在新常态下我市发展速度有所放缓，但基本面持续向好，发展基础不断夯实，城市品位和美誉度不断提高，居民收入增幅居全区前列，就业保持稳定，物价总体平稳，人民安居乐业，桂林正呈现蒸蒸日上的良好发展势头。成绩的取得来之不易，是市委总揽全局、科学谋划的结果，是市人大、市政协有效监督、大力支持的结果，是全市人民攻坚克难、共同奋斗的结果。在此，我代表市人民政府，向全市人民，向人大代表和政协委员，向各民主党派、工商联和无党派人士、各人民团体以及社会各界人士，向中央、自治区驻桂单位、驻桂部队和武警官兵，向关心支持桂林建设发展的港澳台同胞、海外侨胞和国际友人，表示衷心感谢并致以崇高的敬意！

　　在充分肯定成绩的同时，我们也清醒地看到存在的困难和问题：驾驭新常态下经济发展的能力有待进一步提高，应对经济下行压力的思路办法不多，发展中不平衡、不协调、不可持续问题仍然突出。工业缺乏大项目、大产业、大园区支撑，部分企业生产经营困难，增长动力不足。现代服务业整合提升和服务管理相对滞后，龙头企业少，带动作用不够强。资金、土地、环境等制约因素仍较突出，加上桂林特有的经济发展受限因素及繁重的民生保障任务，我市改革和发展任务十分艰巨。对于这些问题，我们要高度重视，采取有力措施，切实加以解决。

　　二、2015年工作安排

　　各位代表，2015年是实施“十二五”规划的收官之年，也是桂林国际旅游胜地建设中期目标实现之年，做好今年工作，承上启下，意义重大。今年政府工作的总体要求是：深入贯彻党的十八大、十八届三中、四中全会和习近平总书记系列重要讲话精神，以邓小平理论、“三个代表”重要思想、科学发展观为指导，全面贯彻落实自治区党委、政府及市委各项决策部署，坚持稳中求进工作总基调，坚持以提高经济发展质量和效益为中心，坚持总量提质、分量提速，坚持桂林国际旅游胜地建设“一本蓝图绘到底”，主动适应经济发展新常态，按照“加快建设新城，着力提升老城，产业融合发展，城乡协调推进，生态文化相融，富裕和谐桂林”既定思路，狠抓改革攻坚，突出创新驱动，着力工作落实，切实保障和改善民生，加强法治政府建设，促进经济平稳健康发展和社会和谐稳定。

　　按照上述总体要求，综合考虑各种因素，今年经济社会发展主要预期目标是：地区生产总值增长8%；组织财政收入增长8%；全社会固定资产投资增长18%；社会消费品零售总额增长13%；城镇居民人均可支配收入增长9%，农民人均纯收入增长10%；城镇化率达到47%；居民消费价格涨幅控制在3.5%以内；城镇登记失业率控制在4.5%以内；市区空气中PM2.5[4]、PM10[5]年均浓度分别下降6%和7%；主要节能减排指标完成自治区下达目标。提出以上目标，既是适应经济新常态下增速换挡、结构优化、动力转换的规律趋势，又考虑到桂林作为一个知名城市和县域大市，要改变发展滞后的状况，必须达到或略高于全国全区的发展水平。确定这样的目标，有发展基础，有政策优势，有项目支撑，有要素保障，兼顾了需要与可能，通过努力是可以达到的。我们要充分认识新常态下的新趋势、新特征、新动力，坚定信心、搭好平台、建立纽带，适应新常态、打造新亮点，把我市发展优势转化为发展实效，切实以转型发展来实现赶超跨越，努力走出一条新常态下具有桂林特色的创新发展之路。

　　要实现全年工作目标任务，必须抓好以下重点工作：

　　（一）以胜地建设为引领，全面实施产业带动战略

　　坚持桂林国际旅游胜地建设“一本蓝图绘到底”，围绕完成《规划纲要》中期目标，特别是对三次产业发展提出的目标要求，把产业发展摆在更加优先的位置，以转方式调结构为主线，以产业高端化、高质化、高效化为主攻方向，构建符合桂林实际的现代产业体系，不断夯实胜地建设产业基础，力争实现胜地建设中期目标。

　　强力发展现代工业。做大做强桂林经济，提高财政收入增速和质量，保持新常态下经济发展的动力和活力，必须强化工业的支柱地位、引擎作用和保障功能，必须毫不动摇地把工业经济增量提质摆在更加突出的位置，一手抓存量提升，一手抓增量扩张，着力培育大园区、大项目、大企业、大产业，开创现代工业发展新局面。强化政府对企业的有效服务，把为企业排忧解难作为各级政府和部门的应尽之责，成为抓经济工作的新常态。进一步畅通企业信息渠道，建立服务企业的责任制度、协调制度、通报制度和绩效评估制度，在企业发展规划、技术创新、品牌宣传、要素保障等方面，分门别类地为企业出谋划策，提供有效服务。发挥桥梁纽带作用，建立稳定实用的政银企保互动平台，切实帮助有产品和市场优势的企业解决融资增信问题。

　　创新园区建设管理机制，加快园区提档升级，推动产业集约集聚发展。继续落实高新区扩区强权政策，全面启动临苏经济开发区建设，打造高新区和临苏经济开发区东西两翼齐飞的产业发展新格局。更新园区建设理念，放开园区建设主体，引入更多市场要素，全方位进入园区基础设施建设、产业发展、配套服务及园区运营等领域。加快制定有利于园区基础设施建设和运营、有利于产业引进和产业链培育、有利于功能配套和要素集聚的园区发展政策措施，探索充满活力的园区管理和运营模式，支持园区发展基金培育，推动产城互动发展、市区和县域园区互动发展。重点推进苏桥经济开发区、秧塘工业园和荔浦、灵川、兴安、平乐4县工业集中区进行园区企业化管理、市场化运作试点，推动全州、兴安和灌阳产城互动试点园区建设见成效，力争全市工业园区完成工业总产值1500亿元。

　　加快产业结构调整，着力培强重点产业，提高重点产业延伸度。先进装备制造业方面，重点促进福达集团年产25万根发动机曲轴等一批项目竣工投产，推进北汽集团与汽车配件生产企业合作、广州万力集团重组中化橡胶（桂林）公司，推动客车产业重组优化；加快高端数显量具量仪和特种数控机床及自动化设备产业化等项目建设；加大模具产业引进力度，做大做长上下游产业链。生物医药方面，继续做大做强科伦药业、澳林制药等医药企业，加快三金药业扩能搬迁和中药城等项目建设，新开工花江生态科技园。电子信息方面，加快建设国家新型工业化（电子信息）示范基地，加快中电科光通信产业园、电工电子新材料产业基地建设，新开工广州白云电气的电气节能及电力电子产业基地等项目，启动“中国东盟信息交流中心及沃易购电子商务基地”桂林备份中心项目，逐项落实我市与国家信息中心合作的具体项目，尽快建立智慧产业研究院，加强北斗技术研究与产品开发，推动北斗产业发展，确立我市相关信息服务产业发展的前沿地位。同时，加快漓泉科研中心等生态食品方面的项目建设。加快培育新兴产业，加大扶持力度，以产业引进、技改投入和自主创新为重点，推动一批创新能力强、特色突出的战略性新兴产业示范基地建设，推动新材料、新能源、节能环保等新兴产业突破发展。

　　以智能制造为突破口，推进两化深度融合。加快创建国家两化深度融合试验区，加强信息网络公共基础设施建设。大力发展工业互联网，在食品、医药等领域开展试点示范，培育智能检测、全产业链追溯等工业互联网新模式。以瀚特物联网“新三板”挂牌为契机，加快物联网技术研究与产品开发、云计算及信息服务业发展。加快推进信息惠民、信息消费国家试点城市建设，持续开展“宽带桂林”建设，培育发展基于互联网的信息消费。继续推进抓大壮小扶微工程，鼓励引导企业上市融资、组建产业联盟，鼓励企业利用电子商务平台，拓宽营销渠道，提升市场开拓能力，鼓励企业争创主席质量奖、市长质量奖，以质量打造核心竞争力，力争实现产值超亿元企业达460家，四大重点产业及新兴产业比重持续提升。

　　提速发展以旅游业为龙头的现代服务业。按照“世界水准、国际一流、国内领先”的标准，加快推进旅游产业转型升级。实施旅游国际化战略，加快推进旅游形象、营销、产品、功能、服务、管理国际化。创建“中国国际特色旅游目的地”品牌，开展“桂林城市整体形象品牌塑造与营销”行动，设计制作与国际接轨、体现桂林文化特色的视觉识别品牌营销系统。引进国际化经营理念和管理方式，鼓励国际资本和知名品牌进驻桂林。继续加快建设一批“高大上”旅游项目，加快推进万达文化旅游城、阳朔瑞盛旅游休闲世界、“玉圭园·环球名胜”旅游综合体二期、全州天湖国际高山生态度假旅游区和阳朔巅峰国际投资人度假区、桃花江旅游度假区等重大项目，推动“夜王城”等一批旅游演艺项目建设，提升休闲旅游项目的数量和品质。继续加强与海航集团合作，实现桂林航空公司正式运营。加快推进旅游与相关产业融合发展。着力打造浪漫温情游等十大旅游产品[6]，重点开发高铁旅游、低空旅游、体育旅游、体验旅游等旅游新业态，重点建设一批以休闲度假酒店、休闲街区、休闲景区、民居旅馆、星级农家乐等为主体的特色休闲旅游产品集群。深化阳朔、兴安等特色旅游名县建设，推动龙胜等特色旅游名县创建工作，扩大永福等长寿之乡的影响力，加快各县区特色旅游资源整合，实施产品开发、宣传促销、标准化管理的统筹推进，打造县域旅游升级版，培育旅游新增长极，实现全市旅游总收入增长22.3%，旅游外汇收入增长6%。

　　优化提升旅游发展环境。强力推进旅游基础设施和公共服务设施建设，重点在桂林北站等区域规划建设游客中心，完善旅游换乘接驳、信息咨询服务等功能，深入实施旅游厕所建设提升示范工程，新建改造旅游厕所180座。加大胜地政策实施力度。重点抓好两项落地免签政策宣传营销和国际航线培育，落实各项配套措施，使更多境外游客享受政策的便利；落实个人赴台自由行等政策；继续争取国家、自治区层面对桂林的政策支持。依法整治旅游市场秩序，完善旅游投诉统一受理平台，严厉查处无资质经营、无资格执业、强迫购物、诱导欺骗游客等违法行为，规范旅游企业经营，培育市场主体，规范提升漓江游览，净化旅游环境。

　　全面提升现代服务业规模和水平。加强对分散的商业业态进行行业或区域整合，实行统筹管理，实现服务业规模提升。全面实行服务业标准化管理，积极组织第二批服务业标准化试点单位开展工作，大力实施桂林米粉店质量星级评定及改造提升工程，实现服务业品牌提升。发挥龙头企业的带动作用，实现服务业发展动力提升。开工新建漓东中央商务区（CBD）项目，持续推进高新万达广场建设，确保国庆节前建成营业；全面建设华润万象城、海吉星食尚港等一批城市商业综合体，重点推进信和信·桂林国际智慧健康旅游产业园、龙光长寿养生谷、崇华中医街等健康养生项目。推进创意产业园、保利文化产业园等项目建设，大力发展文化创意、网络服务、数字动漫等新型文化产业。推进电子商务、第三方物流、连锁经营等现代流通业发展，加快城北商贸物流园区、临桂新区物流配送中心、桂林物流公共信息服务平台等项目建设。加强网络营销，深化与淘宝、百度和爱奇艺等网络平台合作，重点推进高新区电商产业等项目建设，打造桂林“电商谷”，推动现代服务业转型升级。重视体育产业发展，与法国阿讷西市合作办好阳朔国际越野挑战赛。积极筹办第九届联合国世界旅游组织／亚太旅游协会旅游趋势与展望国际论坛、中国—东盟博览会旅游展、第五届桂林国际山水文化旅游节、第六届中国桂林国际旅游博览会、第三届中国（桂林）国际健康养生服务产业创新发展高端论坛等重大节事活动及县域特色节庆活动。培育引导发展型和享受型消费热点，落实职工带薪休假制度，提升旅游休闲消费，鼓励养老健康家政消费，继续组织多种形式的大型促销活动，多措并举促进消费增长。

　　加快发展现代特色农业和绿色农业。立足桂林作为广西农业大市的产业基础和比较优势，坚持“绿色+特色”理念，实施“品牌＋冷链＋营销”战略，以农民增收为目标，在稳定粮食生产的前提下，经过今明两年努力，基本建成20～30个自治区级和市级现代特色农业（核心）示范区，打造一批优质农产品品牌，形成一批特色农家乐集群，培育一批职业农民和种养大户，提升一批运作规范的农民专业合作社，壮大一批农产品精深加工龙头企业。

　　打造特色效益农业品牌。扎实推进桂林国家农业科技示范园区建设，使之成为引领现代农业的标杆。大力推广农业清洁生产技术，加强“三品一标”[7]工作，争创全国农产品出口质量安全示范市，办好广西名特优农产品交易会，提升金桔、荔浦芋、月柿、砂糖桔、罗汉果、金槐等优势品牌成为国内著名品牌，打造葡萄、红提、食用菌等一批自治区级品牌，进一步提升优势水果、特色蔬菜、中药材生产能力，使总量、人均占有量继续领跑全区。加快发展东山猪、竹狸、禾花鱼、冷水鱼等特色养殖业，推进南方现代草地发展项目，大力发展牛、羊等草食动物养殖。做大做强休闲观光农业。结合现代特色农业（核心）示范区创建，打造一批特色休闲农业景观，充分发挥阳朔、恭城、灵川、灌阳、龙胜等全国休闲农业示范县引领作用，引导农家乐向高品位、特色化、集群式发展，推动乡村旅游全面升级。

　　实施农业基础提升工程。市本级财政安排专项资金，支持新增100个建制村通水泥（沥青）路和通自然村屯道路200公里以上，完成1500个自然村屯内道路硬化。继续推进400个“普惠制”新村建设，完成200个自然村屯阳光惠农工程。继续实施中小河流治理、水土保持综合治理、农田水利、交通和高标准基本农田建设。全面推进水稻全程机械化，提升农机装备水平。加快发展家庭农场、农民专业合作社等新型农业经营主体，大力发展农产品精深加工，规划建设农产品冷链物流项目，加快发展农产品电商、直销配送、农超对接等现代流通方式，提高“南菜北运”规模水平，在广大农村建立起成本低、效率高、服务好的物流渠道，推行“科学种养+精深加工+现代物流”三位一体发展模式，增强特色农产品市场竞争力。

　　（二）以重大项目为抓手，增强经济发展支撑力

　　扩投资、上项目仍然是桂林经济发展的最大动力，是完成好全年目标任务的“压舱石”。要发扬钉钉子精神，以实体经济和重大基础设施建设为主攻方向，以项目快开工、快竣工、快见效为终极目标，进一步优化投资结构，扎实解决项目建设存在的突出问题。

　　切实加大有效投资强度。以自治区层面推进的重大项目及市领导包抓的20个重中之重项目[8]为重点，努力开辟投资新领域，扩大有效投资规模，年度实施重点项目800项，完成投资746亿元以上。围绕构建完善的现代化基础设施体系抓项目，实施交通、能源、水利、新型城镇化等重大基础设施重点项目288项，完成投资253.5亿元。交通方面，协调推进两江机场T2航站楼建设；加快建设高铁配套场站，争取开工桂林铁路西货运中心，做好桂林动车所前期工作；实现灌阳（永安关）至全州（凤凰）高速公路建成通车，完成阳朔至鹿寨高速公路主体工程，加快资源至兴安、桂林至三江高速公路项目建设，力争开工建设荔浦至玉林高速公路，强力推动灌阳经恭城至平乐高速公路的招商建设。能源方面，加快推进临桂新区分布式能源项目，开工建设兴安石板岭、严关及全州青山口等风电场项目。水利方面，重点推进桂林市防洪及漓江补水枢纽工程建设，实施72座病险水库（水闸）除险加固。围绕打造富有桂林特色的现代产业体系抓项目，推进重点产业项目425项，完成投资455.8亿元。围绕构建基本均等的公共服务体系抓项目，实施教育、文化、卫生等社会公益重点项目71项，完成投资29.1亿元。围绕发展生态经济、建设生态文明抓项目，推进生态环保项目16项，完成投资7.69亿元。发挥财政扶持资金、产业发展资金及各类发展基金的引导作用，撬动更多社会投资向战略性新兴产业、现代服务业、现代农业等实体经济聚集。

　　切实加快项目建设进度。进一步完善“联审联批”“五个一”“三清单一问责”等重大项目推进机制，提升重大项目建设的速度、质量和效益。强化项目保障机制，对重点建设项目特别是重大产业项目，在用地指标、发展资金、能耗总量、环境容量、政策扶持上给予倾斜。强化工作考核机制，建立健全重大项目工作考核评价体系，确保按照“路线图”和“进度表”完成投资计划。坚持问题导向，实行“三线并进”机制，同步开展项目政策处理、审批、供地等环节前期工作，推进项目落地终端前移，实现早开工、早建设、早竣工、早投产。抓紧做好“十三五”规划项目策划、研究和储备，争取更多项目纳入国家和自治区投资计划。

　　切实加大招商引资力度。创新营销方式，成立营销委员会，抓好旅游营销、产业营销、城市营销。以高铁经济带建设为契机，围绕重点产业和新兴产业，对接港澳台、珠三角、长三角等重点区域，瞄准全球500强、国内500强企业，按照技术关联、价值关联、配套关联的要求，进行精准招商，强化产业链招商，着力引进一批投资规模大、产业关联度高、带动能力强的项目。健全市场化招商引资机制，加强市县区、政企联动，发挥工商联资源广泛的优势，加强与境内外工商界、商会交流，积极推进联动招商；探索以企业化组织为主体的招商引资模式，聘用投资促进顾问，推进和规范委托招商。力争引进区外境内到位资金495亿元，实际利用外资超4.2亿美元。

　　（三）以提升名城品质为目标，提高城乡建设治理水平

　　坚持走以人为核心的新型城镇化之路，围绕打造品位休闲之都、健康美丽浪漫之城的目标，完善功能、突出特色、彰显文旅、提升品位、创新环境，高度重视人民群众普遍关心的基础设施改善、环境质量优化等问题，更富实效地推进城市综合治理，提升城乡管理水平。

　　加快临桂新区和各县新城建设。围绕满足“入驻入住”条件，立足功能配套和产业发展，带动新区人气、景气、商气的集聚，加快桂林广电大厦、桂林日报社传媒中心后续建设并投入使用，加快建设市政道路桥梁基础设施，力争西城大道、宏谋大道、万平路桥等13个在建节点路桥项目全面完工，路灯、绿化同步跟进，完成湖塘水系项目主体工程，启动建设临桂新区北区水系、沙塘大道、花园路等基础设施项目。高度重视新区民生需求，全面建设新区医院、学校、公园、菜市场、商场等公共服务项目，完善供水、供气、供电、环卫、排水、防洪等市政管网设施，增强新区生活服务配套能力。扎实抓好保利文化产业园、凤凰旅游休闲度假区等产业项目建设，增强新区发展动力，打造功能更完善、环境更优美、都市气息更浓厚、聚集辐射能力更强大的现代新型城区。建设桂北、桂南副中心城市，统筹谋划桂灵都市区[9]，加快全州、荔浦、阳朔、灌阳、平乐等县城新区建设，增强县城综合承载力。

　　加快老城疏解提升步伐。继续实施“南畅、北通、东拓、西联”工程，力争完成国道321阳朔至桂林段扩建工程全线征地拆迁和路基建设，力争完成桂黄公路灵川段提升改造工程、西二环路北辰立交、中隐路西延长线等重大路网项目，基本建成福利路二期快车道，打通纵贯新区老城的南北通道；全面开建万福东路和龙门大桥，确保2016年建成使用，实现东西交通路网大连接。启动火车北站西区场站及相关路网建设，加快形成连接航空、铁路、公路的立体综合便捷交通网络。加强重要交通节点改造，启动香江饭店路口立交桥等项目建设，启动主次干道和背街小巷改造工程，加快汽车总站搬迁。深入实施棚户区改造项目，重点改造城北福利路铁路片区、漓江东岸福隆园和塔山片区等棚户区。规范夜市管理，全面提升老城形象。加快各县老城改造进程，完善交通路网、公共基础设施，实施城乡风貌改造，塑造生态文化景观，优化发展空间，凸显县城特色。高标准、大力度建设第二批新型城镇化示范乡镇，扎实抓好城镇建设“百镇示范工程”和“广西特色名村”创建工作，打造一批“区内一流、全国知名”的特色城镇。

　　加强城市综合治理。建立健全城市综合治理长效机制，深入推进环境卫生、违法占道经营、交通秩序、大气污染、漓江上游生态环境五大专项治理行动。加强城市管理的舆论监督。制定实施城市管理微制度，切实推进停车场、集贸市场、垃圾转运站、公共厕所等市政设施建设，着力推进管理创新，探索完善网格化管理、市场化服务、门前“三包”、渣土管理以及背街小巷、城中村、无物业管理小区、城乡结合部综合整治的新制度和新举措。加快智能交通建设，打好交通治堵持久战，采取联通路网、渠化节点、精细管控、严管重罚、增加停车泊位等综合措施，缓解交通拥堵；倡导绿色低碳出行，优化发展公共交通和公共自行车服务系统，加强出租车行业管理，加速提升交通便利性和可达性。打好清理整顿“违法占地”“违法建设”攻坚战，持续保持高压态势，对新的违法建筑零容忍、零补偿、强拆除。加快“智慧城市”建设，整合利用城市管理资源，提升城市科学化、精细化、人性化管理和服务水平。实施“文明市民工程”，大力开展“改陋习、美桂林、我先行”主题实践活动和“我参与、我奉献、我快乐”文明劝导活动，提升市民文明素质。

　　（四）以生态经济为导向，全面推进生态文明建设

　　顺应广大市民和游客对良好生态环境的新期盼，始终坚持绿色发展、低碳发展理念，大力发展生态经济，呵护好甲天下的桂林山水，让市民和游客喝上洁净的水，呼吸清新的空气，吃上健康的食品，享受绿色的空间。

　　强化生态环境保护和治理。坚持源头严防、过程严管、后果严惩，严格环境保护监管、执法，落实生态环境保护责任制，划定生态保护红线。突出抓好水环境质量改善，积极推进全国水生态文明城市建设试点工作，全面实行最严格水资源管理制度考核。实施漓江综合治污工程，重点抓好漓江及其支流环境综合整治，力争完成漓江市区段、灵川县段所有排污管网改造和清风沟、小东江、瓦窑河、南溪河、灵剑溪、桃花江等支流截污工程，城镇污水处理率92%以上。加强漓江、湘江等流域水源林保护和修复，继续强化青狮潭水库整治和生态建设，全面改善漓江水质，加快城市备用水源建设。加强会仙湿地生态保护。突出大气环境治理，加大大气环境保护执法力度，重拳整治采石场、小砖厂、施工工地及道路扬尘污染，全面清理改造燃煤小锅炉，加快淘汰老旧车辆和“黄标车”，使空气质量优良天数有所增加。推进城镇垃圾分类处理，提升医疗废弃物处理能力，生活垃圾无害化处理率100%。加强土壤污染、重金属污染、石漠化治理。推进低碳城市试点工作，加快雁山区低碳经济产业示范园建设，创建临桂新区绿色生态城区，全面推动清洁能源应用。突出抓好节能减排，严格实施污染物排放总量控制，完成“十二五”节能减排任务。

　　大力发展生态产业。坚持生态建设产业化、产业发展生态化，加强生态产业研究，大力发展生态工业、生态农业、生态旅游业和林业生态经济，加快构建新型生态产业体系。充分利用桂林生态优势、人才技术优势，找准切入点，力争在生态食品和节能环保产业、废弃物处理和再生资源回收利用、大气和水污染治理等方面走在全区前列。加快园区循环化改造，促进传统优势产业提升。大力推动经济生态文化融合发展，创建一批生态文化教育基地，推进建设一批生态文化基础设施，创作一批生态文化作品，培育一批生态文化企业，着力打响“秀丽山水、生态桂林”的生态文化品牌，推动绿色经济、有机经济、循环经济一体化发展，生态人居、生态环境、生态文化同步建设，使生态优势转化为经济优势。

　　继续推进“美丽桂林·生态乡村”建设。全面开展“村屯绿化”“饮水净化”“道路硬化”专项行动，营造“一个村屯一座绿岛”的生态美景，提升村屯自来水普及率，加快开展千人以上农村集中供水工程饮用水水源保护区划定工作，有效解决农村群众出行难和安全饮水问题。每县区建设一个“三化”融合示范带，重点推进大圩—草坪—兴坪漓东百里生态示范带建设，全面启动中国传统村落的保护和开发工程，加快打造一批与国际旅游胜地相匹配的生态乡村。

　　（五）以改革开放为动力，深入实施创新驱动发展战略

　　改革就是要解决发展中的问题。要坚持问题导向，大力推进改革创新，不断扩大开放，以更有力的措施，为各类市场主体营造宽松有序的发展环境，为新常态下桂林的发展拓展新空间。

　　积极推进重点领域改革。不断完善配套政策措施，强化已出台改革方案的落地实施，重点推进行政审批和管理、财税、投融资、金融、土地利用等改革，力破资金、土地、环境等要素瓶颈。继续取消和下放一批行政审批事项，进一步为基层、企业和各种市场主体松绑放权，规范行政审批行为，推行一口受理、限时办理、规范办理、透明办理，完善行政审批事项目录管理，做好“接、放、管”。深入实施商事制度改革，推行“先照后证”。积极推进行政审批标准化建设，完善四级政务服务体系，在全市全面深入实施行政审批“马上办”工作机制；加快建立网上审批服务大厅，推行审批事项网上咨询、网上申请、网上办理和联合审批，使行政审批再提速、办事效率再提升，力破环境瓶颈。全面实施新的《预算法》，完善市县区财税利益分享机制和激励机制，优化财政资金分配，集中财力办大事；积极消化存量负债，防范和控制债务风险；创新筹融资思路，启动“442”融资模式改革，银行贷款达到40%左右，股权、债权、基金和资产证券化融资达到40%左右，采取PPP[10]、TOT[11]和争取上级政府资金等方式融资达到20%左右，力争通过政府直接融资200亿元以上，带动1600亿元以上社会资本；创新金融服务方式，引导金融机构盘活存量、用好增量，优化信贷结构；加快资产经营和资本运营改革，盘活各类资源、整合各类资产，组建相应的产业发展平台公司，建立资产资本运营平台，支持引导信贷投放向重点领域、重点产业、重点项目倾斜，加快开展漓江基金、海航基金、城市发展基金的设立、运营工作，力破资金瓶颈。用好用足旅游用地政策，建立集约节约用地机制，强化土地利用和供给管理；做好自治区和市统筹推进重大项目、重要民生项目、重大产业项目用地保障；通过加快征地、盘活存量用地等办法，解决供地率低的问题，确保每年平均供地率达60%以上；推进永久性基本农田划定和设施农用地的规范管理工作；建立建设用地计划指标执行动态管理机制，对久拖不决、长期延宕的用地项目实行“退出机制”，收回与结余的指标统筹调剂，另行安排使用；大力开展土地收储工作，力争市本级土地收储面积累计达到1万亩以上，力破土地瓶颈。

　　统筹推进国资国企、价格、统计、养老保险等其他改革。继续加大国有企业改革重组力度，积极推进党政机关与企业脱钩，大力发展混合所有制经济。建立非公有资本负面清单制度，按照“非禁即入”原则，放宽资本投资准入，促进非公经济发展。全面推进服务业统计及投资统计制度改革，完成阳朔县、象山区试点工作，建立统计联席会议制度。着力推进价格改革。落实机关事业单位养老保险制度改革措施，同步完善工资制度，政策向低职务人员倾斜，向条件艰苦的偏远乡镇和长期在乡镇工作的人员倾斜，实行乡镇工作补贴，在县以下机关建立公务员职务与职级并行制度。进一步提高村干部待遇。

　　积极推进开放型经济发展。抢抓湘桂、贵广高铁相继开通带来的新机遇，主动融入贵广高铁经济带建设，积极参与湘桂高铁区域合作；加紧调整优化高铁沿线产业布局，加快推动与高铁沿线城市和地区产业发展互补互利、基础设施互联互通、线路政策互推互惠、生态环境联防联治。充分利用高铁带来的“同城效应”，提升商业业态，扩大购物休闲、旅游商务、餐饮住宿等消费的辐射半径；加强铁路、公路、水路、机场等各种交通运输方式衔接，实现高铁与旅游目的地无缝对接，着力打造高铁经济带核心城市。积极拓展与欧美日韩澳新等国家和地区交流合作，深化与张家界等国内友好城市的合作，主动融入珠江—西江经济带，争取自治区对桂江基础设施建设给予更多支持。加大口岸建设力度，积极创建国际卫生机场。加快实施加工贸易倍增计划，优化外贸结构，加强重点企业扶持，引导优势企业“走出去”，实现外贸进出口总额增长5%。

　　深入实施创新驱动发展战略。全面整合资源，推进“大研发”体系建设。建设桂林自治区级自主创新示范区，实施“1020”创新驱动计划[12]，遴选实施一批重大攻关项目和重点应用项目，突破一批制约产业发展的关键技术，支撑重点产业和战略性新兴产业发展。加强工程技术研究中心等研发平台、大学科技园等科技企业孵化器建设，加快构建以市场为导向、企业为主体、高校院所共同参与的协同创新体系。进一步加强与“985”高校合作，充分发挥驻桂高校和科研院所作用，促进科技成果转化和产业化，将科教优势转化为经济发展优势。开展全民发明创造活动，继续实施发明专利倍增计划，力争每万人拥有发明专利达到3.5件。加强知识产权保护和运用。加快建设桂林国家级文化和科技融合示范基地，培育一批文化和科技融合示范企业，建设工艺美术产业示范园。加强环境保护、节能减排、人口健康等领域的技术开发与应用示范，让科技成果惠及广大民众。高度重视招才引智工作，加强漓江学者、人才小高地等重大人才工程建设，营造科技人才创新创业的良好环境。积极创建“全国质量强市示范市”。

　　（六）以改善民生为根本，促进社会事业全面发展

　　民之所望，施政所向。我们必须始终把保障和改善民生作为政府工作的出发点和落脚点，作为政府公务人员履职担责的检验标尺，真心实意解民忧、谋民利、惠民生，努力让全市人民的日子越过越红火，生活越来越幸福。

　　强化就业创业和社会保障。全市安排就业与社会保障投入25.2亿元，增长22.9%。深入开展“就业援助月”“春风行动”等系列活动，加强高校毕业生、农村转移劳动力和城镇困难人员就业工作。加强职业技能培训，积极推动大众创业，多渠道增加就业岗位，确保全年城镇新增就业6.3万人以上，城镇失业人员再就业1.5万人以上。多渠道促进城乡居民增收，重点提高中低收入居民的收入水平。建立健全社会保障与就业联动机制，积极推动各类单位和人员参保，新农合、城乡居民养老保险参保率完成自治区下达任务；进一步提高城乡低保、保障房建设、农村五保供养、医疗救助、孤儿救助、自然灾害和临时困难救助水平和保障标准，规范完善社会救助体系。逐步推进农民工市民化和常住人口基本公共服务全覆盖，加快基本公共服务均等化，让广大市民的生活更有保障、心里更加踏实。

　　扎实做好精准扶贫。扶贫开发是头号民生工程，实施精准扶贫是打好新一轮扶贫开发攻坚战的有效举措。加快贫困群众脱贫步伐，力争减少贫困人口6万人以上。强力推进扶贫生态移民工程，加快实施“雨露计划”，全面落实扶贫对象帮扶到户，加大金融、产业、旅游、教育、科技扶贫力度，增加财政专项扶贫资金，加快石漠化片区、少数民族地区、边远山区扶贫攻坚步伐，继续开展整村推进扶贫开发工作，使贫困地区尽快破穷障、拔穷根、摘穷帽，早日过上小康生活。

　　推动教育优质均衡发展。全市安排教育投入48.9亿元，增长13.4%。继续实施教育发展十大重点工程，建立市教育发展基金，实施第二期学前教育三年行动计划，推进乡镇幼儿园、多元普惠幼儿园建设，全市学前教育三年毛入园率86%以上，抓好义务教育学区制改革试点，新建14所中小学校，加快义务教育均衡发展，全市九年义务教育巩固率95%以上，实施普通高中教育特色发展工程，全市高中阶段教育毛入学率90%以上，全面深化职业教育综合改革，积极引进社会资本，大力发展职业教育，继续支持普通高校发展，加快推进桂林师专临桂新校区建设，让更加优质、更加公平的教育资源惠及全市人民。

　　统筹推进卫计事业发展。深化医药卫生体制改革，重点加强基层医疗、儿童医疗、重大疾病防治设施等卫生计生服务体系建设，推进卫生信息化建设，力争实现市直医疗机构门诊一卡通服务，打造桂林“崇华中医”品牌。实施好单独二孩政策，巩固提升诚信计生工作。鼓励引导社会办医、医养结合、构建多元办医格局，切实缓解看病难、看病贵问题。

　　大力发展文化体育事业。深入实施“寻找桂林文化的力量，挖掘桂林文化的价值”工程，全面加强文化资源调查研究和保护利用，继续支持灵渠申遗，做好名人故居的保护修缮和开发利用工作，推进历史文化标识和非物质文化遗产展示馆建设，加快甑皮岩、靖江王陵两个考古遗址公园和“飞虎队”桂林遗址公园建设，承办2015年国家考古遗址公园联盟联席会议；开展纪念抗日战争胜利70周年系列活动，举办“新西南剧展”，传承抗战文化精神。深入实施文化惠民工程，完成年度村级公共服务中心、“村村通”广播电视无线发射台站建设任务，推进“一院两馆”正式对外开放，持续提升“百姓大舞台”“漓江之声”“百姓大讲坛”“读书月”“周末大家乐”等文化品牌影响力。加强健身场地建设，深入开展全民健身运动，提升竞技体育水平，更好地满足人民群众多层次、多样化文化体育需求。

　　加强和创新社会管理。创新社会管理模式，畅通市民诉求通道，改进公共服务方式。建立和完善大调解机制、突发应急处置机制，持续开展矛盾纠纷大排查、大接访、大调处、大防控活动，进一步落实社会稳定风险评估机制，创新化解机制，强化源头预防。深化平安桂林建设，继续开展社会治安综合整治活动，不断强化反恐维稳工作，持续抓好“扫黄打非”，确保社会治安良好稳定。强化责任落实，抓好安全生产能力建设，强化安全隐患排查治理和安全专项整治。完成“六五”普法任务，推动诚信桂林建设。促进民族团结进步，强化少数民族地区基础设施建设，办好资源县享受自治县待遇20周年县庆活动。全面做好国防动员、民兵预备役、人民防空和双拥共建工作。

　　实施重点民生工程。坚持民生优先，多渠道筹措资金，在确保完成自治区下达我市重点民生项目任务的同时，年度计划投资57亿元，重点实施漓江排污综合治理、城市棚户区改造、旅游公厕建设提升、背街小巷系统改造、城市内涝治理、城市交通节点改造、公共交通系统建设、新农村建设、学校新建改建、公共服务便民项目等涵盖17县区的市级十大重点民生工程，切实办好群众期待的实事好事。

　　三、深入推进依法行政，加快建设法治政府

　　贯彻依法治国基本方略，以建设职能科学、权责法定、执法严明、公开公正、廉洁高效、守法诚信的法治政府为切入点，加强制度创新、流程重构、机制改革、文化重塑，加快构建“六位一体”政府运行模式，全面提高政府治理现代化水平。

　　（一）依法全面履行政府职能。加大法治宣传力度，强化依法行政意识，坚持法定职责必须为，法无授权不可为，坚决纠正不作为、乱作为，严厉惩处失职、渎职。探索制定行政权力清单、责任清单和负面清单，用政府权力的“减法”，换取市场和社会活力的“加法”，充分发挥市场在配置资源中的决定性作用，更好发挥政府作用，实现“全能政府”向“有限政府”转变。加强事中事后监管，坚决惩处假冒伪劣、蓄意污染环境、危害食品药品安全等行为，建立科学的抽查制度、责任追溯制度、经营异常名录和黑名单制度。培育和规范中介组织。强化新常态下对经济运行的监测和预警。

　　（二）强化行政依法决策。贯彻落实公众参与、专家论证、风险评估、合法性审查、集体讨论决定的重大行政决策法定程序。加强新型智库建设，成立重大行政决策专家咨询论证委员会，大力推行咨询问政；建立网络问政平台，健全部门之间、部门与社会组织之间协同工作机制。落实重大行政决策终身责任追究制及责任倒查机制，确保决策制度科学、程序正当、过程公开、责任明确。加大政府重大投资项目、民生项目和重大政策落实情况跟踪等方面的审计力度，加强对审计问题整改的跟踪，突出督促检查。

　　（三）严格规范公正文明执法。按照减少层次、整合队伍、提高效率的原则，合理配置执法力量，减少市县两级政府执法队伍种类，加强市县两级政府行政执法体制改革，理顺行政强制执行体制，组建城市管理综合行政执法局，构建城市管理统一综合执法体制，提高执法和服务水平。严格实行行政执法人员持证上岗和资格管理制度，健全行政执法裁量权基准制度，加大对劳动保障等关系群众切身利益的重点领域执法力度。

　　（四）强化行政权力制约和监督。健全依法行政考核机制。自觉接受人大及其常委会的法律监督和人民政协的民主监督，坚持重大决策出台前向人大报告。健全政府部门与民主党派对口联系工作机制，不断发展和完善政府协商民主。认真听取各民主党派、工商联和无党派人士的意见和建议，及时办理人大代表建议和政协提案。充分发挥行政监察等专门监督的作用，高度重视发挥新闻媒体和社会公众的监督作用。强化规划引导，科学编制“十三五”规划。

　　（五）全面推进政务公开。重点推进行政审批、财政预算决算和“三公”经费、保障性住房、价格和收费、征地拆迁、投资贸易、招投标等重点领域的政府信息公开，以及以教育、医疗为重点的公共企事业单位办事公开。加强电子政务、便民服务和基层政务服务平台及公共资源集中交易平台建设，实现政务服务与公共资源交易一体化管理，确保权力规范、阳光运行。

　　（六）加强廉洁政府建设。继续推进党风廉政建设和反腐败斗争，认真贯彻中央八项规定精神，控制行政成本，完成党政机关公务用车制度改革，确保“三公”经费只减不增，把更多的物力、财力用在群众最迫切需要解决的问题上。严肃财经纪律，倡俭治奢，严格公共资金、公共资源、国有资产监管，认真落实“一岗双责”制度，加大审计、监察工作力度，严肃查处各类违法违纪案件，严厉整治“庸懒散拖”，以公正廉洁的良好形象取信于民。

　　各位代表，桂林正处在深化改革、加快发展的重要阶段，我们的任务光荣而艰巨，我们的责任重大而崇高，我们有信心、有决心、有能力担负起人民赋予的神圣使命。让我们紧密团结在以习近平同志为总书记的党中央周围，在自治区党委、政府和市委的领导下，努力践行“实干就是能力，落实才是水平”的工作理念，克难攻坚，扎实工作，为完成今年和“十二五”规划目标任务，为加快实现桂林“两个建成”目标而努力奋斗!
